# Preferred Treatment with Curative Intent for Left Lateral Segment Early Hepatocellular Carcinoma under the Era of Minimal Invasive Surgery

**DOI:** 10.3390/jpm12010079

**Published:** 2022-01-09

**Authors:** Tsung-Han Wu, Yu-Chao Wang, Hao-Chien Hung, Jin-Chiao Lee, Chia-Ying Wu, Chih-Hsien Cheng, Chen-Fang Lee, Ting-Jung Wu, Hong-Shiue Chou, Kun-Ming Chan, Wei-Chen Lee

**Affiliations:** 1 Department of General Surgery and Chang Gung Transplantation Institute, Chang Gung Memorial Hospital at Linkou, Chang Gung University College of Medicine, Taoyuan 33302, Taiwan; domani@cgmh.org.tw (T.-H.W.); b9002072@cgmh.org.tw (Y.-C.W.); mp0616@cgmh.org.tw (H.-C.H.); b9302012@cgmh.org.tw (J.-C.L.); chengcchj@cgmh.org.tw (C.-H.C.); lee5310@cgmh.org.tw (C.-F.L.); wutj5056@cgmh.org.tw (T.-J.W.); chouhs@cgmh.org.tw (H.-S.C.); weichen@cgmh.org.tw (W.-C.L.); 2 Department of Gastroenterology and Hepatology, Chang Gung Memorial Hospital at Linkou, Chang Gung University College of Medicine, Taoyuan 33302, Taiwan; alicewu0912@gmail.com

**Keywords:** hepatocellular carcinoma, curative treatment, liver resection, laparoscopic surgery, radiofrequency ablation, outcome

## Abstract

Background: Hepatocellular carcinoma (HCC) occurring at the left lateral segment (LLS) is relatively susceptible to treatment with curative intent in terms of tumor location. However, outcomes might vary depending on the selection of treatment modalities. This study aimed to analyze patients who had undergone curative treatment for early HCC at LLS. Methods: A retrospective analysis of 179 patients who underwent curative treatment for early HCC at LLS was performed. Patients were grouped based on treatment modalities, including radiofrequency ablation (RFA) and liver resection (LR). The long-term outcomes of the two groups were compared. Additionally, the impact of the LR approach on patient outcomes was analyzed. Results: Among these patients, 60 received RFA and 119 underwent LR as primary treatment with curative intent. During follow-up, a significantly higher incidence of HCC recurrence was observed in the RFA group (37/60, 61.7%) than in the LR group (45/119, 37.8%) (*p* = 0.0025). The median time of HCC recurrence was 10.8 (range: 1.1–60.9 months) and 17.6 (range: 2.4–94.8 months) months in the RFA and LR groups, respectively. In addition, multivariate analysis showed that liver cirrhosis, multiple tumors, and RFA treatment were significant risk factors for HCC recurrence. The 1-, 2-, and 5-year overall survival rates in the RFA and LR groups were 96.4%, 92.2%, and 71.5% versus 97.3%, 93.6%, and 87.7%, respectively. (*p* = 0.047). Moreover, outcomes related to LR were comparable between laparoscopic and conventional open methods. The 1-, 2-, and 5-year recurrence free survival rates in the laparoscopic (*n* = 37) and conventional open (*n* = 82) LR groups were 94.1%, 82.0%, and 66.9% versus 86.1%, 74.6%, and 53.1%, respectively. (*p* = 0.506) Conclusion: Early HCC at LLS had satisfactory outcomes after curative treatment, in which LR seems to have a superior outcome, as compared to RFA treatment. Moreover, laparoscopic LR could be considered a preferential option in the era of minimally invasive surgery.

## 1. Introduction

Hepatocellular carcinoma (HCC) is the leading cause of cancer-related deaths worldwide. Recent treatment guidelines suggest curative treatment, including radiofrequency ablation (RFA), liver resection (LR), and liver transplantation for very early- and early-stage HCC. Although liver transplantation provides the best survival outcome for these patients, the scarcity of liver donation marks its limitation. Therefore, RFA and LR are still the preferred choices for most of these patients. Many studies have compared the treatment efficacy between RFA and LR; however, the results are still inconclusive [1,2,3].

In the RFA setting, the surrounding liver tissues, including the tumor, need to be ablated to create an adequate margin for curative intent. In such circumstances, avoiding thermal injury and direct puncture to neighboring organs is a major concern. Some procedures have been proposed to avoid these complications, such as image-fusion navigation and artificial ascites [4,5,6,7]. Moreover, the heat-sink effect, bile duct injury, and iatrogenic trans-portal tumor spread are also drawbacks for tumors near the major hepatic pedicles [3,8,9,10]. The shape, volume, and anatomic position of the left lateral segment (LLS) is relatively thin, small, and close to other vital organs, as compared to other parts of the liver. Treating HCC at LLS by RFA will face the problems of thermal injury for subcapsular tumors and heat sink effects/pedicle injuries for deep-seated tumors. These concerns could be avoided by LR, which could completely remove the tumor under direct vision and proper dissection. Apart from that, due to the relatively simple and less anatomical variation of the left lateral segment, left lateral sectionectomy may be considered and is suitable for training surgeons in either the open or laparoscopic approach [11,12]. Therefore, the purpose of this retrospective study was to evaluate the treatment outcomes of RFA and LR for early-stage HCC at LLS.

## 2. Materials and Method

### 2.1. Patients

A total of 1097 patients underwent either LR or RFA as first-line treatment with curative intent between January 2012 and December 2019 at Chang Gung Memorial Hospital Linkou Medical Center (Taoyuan, Taiwan). Among these patients, 179 patients who had received RFA (*n* = 60) or LR (*n* = 119) treatment for HCC located at LLS were included in this study. The medical records of these patients were retrospectively reviewed and analyzed for clinical characteristics, treatment, and outcomes. HCC was confirmed in all patients by pathological examination of specimens obtained from LR or aspiration cytology prior to RFA, as previously described [13]. The study was approved by the Institutional Review Board of Chang-Gung Memorial Hospital, Linkou, Taiwan (IRB:201900441A3), and informed consent from patients was waived because of the retrospective nature of the study.

### 2.2. Diagnosis and Treatment of HCC

Clinically, the diagnosis of HCC was based on the recommendations of the European Association for the Study of the Liver (EASL) and the American Association for the Study of Liver Diseases (AASLD) [14,15]. Arterial phase hyperenhancement with rapid washout on portal-venous and/or delayed phase by contrast-enhanced computed tomography (CT) or magnetic resonance imaging (MRI) were used for diagnosis [16,17].

The decision regarding HCC treatment was made after thorough discussion in the multidisciplinary committee and considering the patients’ desires. Patients treated by LR had complete heart-lung function and liver reserve studies, including indocyanine green retention test at 15 min (ICG-R15). LR was performed by either open laparotomy or laparoscopy, depending on facility feasibility and physician preferences. RFA was performed percutaneously by experienced physicians under proper local anesthesia and intravenous sedation. Complete tumor ablation with an additional margin of at least 1 cm was performed to achieve adequate oncologic treatment.

### 2.3. Follow-Up and Surveillance

All patients underwent regular follow-ups after treatment. Physical examination, liver function test, alpha-fetoprotein (AFP), and hepatic sonography were performed at each visit every 3 months. Image studies, including dynamic CT or MRI, were also arranged every 6–12 months or as needed. HCC recurrence was defined by any forms of HCC detected after curative treatment, and the recurrent patterns included local intrahepatic (contiguous to or within the treated area), distant intrahepatic (in other liver segments or away from the treated area), and systemic metastasis (presence of distant metastasis). Additionally, HCC recurrence was established by typical dynamic image findings or pathology proof for atypical image patterns.

### 2.4. Statistics

Clinical data were compared using Pearson’s chi-square test for categorical variables and analysis of variance for numerical variables. Univariate and multivariate analyses were performed on the patients’ clinical features, tumor characteristics, and treatment methods. Recurrence-free survival (RFS) and overall survival (OS) rates were calculated using the Kaplan-Meier method and compared using log-rank tests. The statistics were calculated using SAS software (Version 9.4) and Stata software (StataCorp. 2015. Stata Statistical Software: Release 14. College Station, TX, USA: StataCorp LP.) A *p* value <0.05 was considered statistically significant.

## 3. Results

### 3.1. Patient Characteristics

The clinical characteristics of the patients are summarized in Table 1. The mean age was higher in the RFA group than in the LR group (*p* = 0.0011). Furthermore, there were more patients with cirrhosis (95.0% vs 62.7%; *p* < 0.001), HCV infection (40.0% vs 20.1%; *p* = 0.0075), smaller tumor size (median size, 2 vs 2.7 cm; *p* = 0.0113), and TNM stage 1 diagnosis (87.9 vs 70.5%; *p* = 0.0129) in the RFA group. A lower platelet count was observed in the RFA group (median, 112 vs. 168 × 10^3^/μL; *p* < 0.0001), which may reflect the degree of cirrhosis. There were no significant differences in AFP levels. HCC recurrence was significantly lower in the LR group (37.8% vs. 61.6%; *p* = 0.0025), in which the RFA group had higher ratio (19.4%) of local intrahepatic recurrence (*p* = 0.0021). Additionally, the median hospital stay was 7 days for LR and 3 days for RFA. Post-treatment complications greater than Clavian-Dindo grade II were 8 (6.7%) and 3 (5.0%) in the LR and RFA groups, respectively. There was no in-hospital mortality in either group.

### 3.2. Survival Outcomes

**Figure 1:**
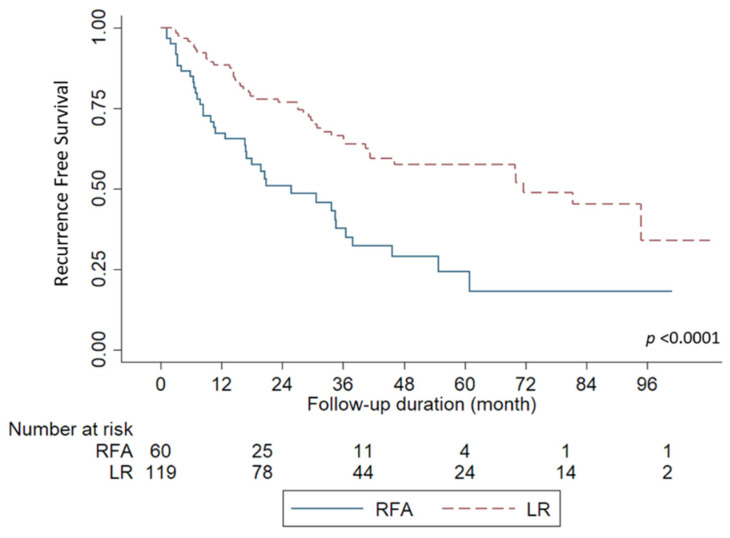
Comparison of recurrence-free survival based on the treatment modalities. Liver resection (LR) showed a significantly better survival curve over radiofrequency ablation (RFA) for patients (*p* < 0.0001).

Overall, the median follow-up period for all included patients was 40.8 months (range, 3.1 to 108.4 months) after curative treatment in this study. During the follow-up period, 36 patients (61.6%) with RFA experienced HCC recurrence, while 45 patients (37.8%) experienced HCC recurrence after LR (*p* = 0.0025). The median time to recurrence was 10.8 months (range, 1.1–60.9 months) in the RFA group and 17.6 months in the LR group (range, 2.4–94.8 months). The Kaplan-Meier survival curves for RFS are shown in Figure 1. The 1-, 2-, 5-year RFS rates for the RFA group were 67.1%, 50.8%, and 24.2%, respectively. In the LR group, the 1-,2-, and 5-years RFS rates were 88.4%, 76.7%, and 57.5%, respectively (*p* < 0.0001).

**Figure 2:**
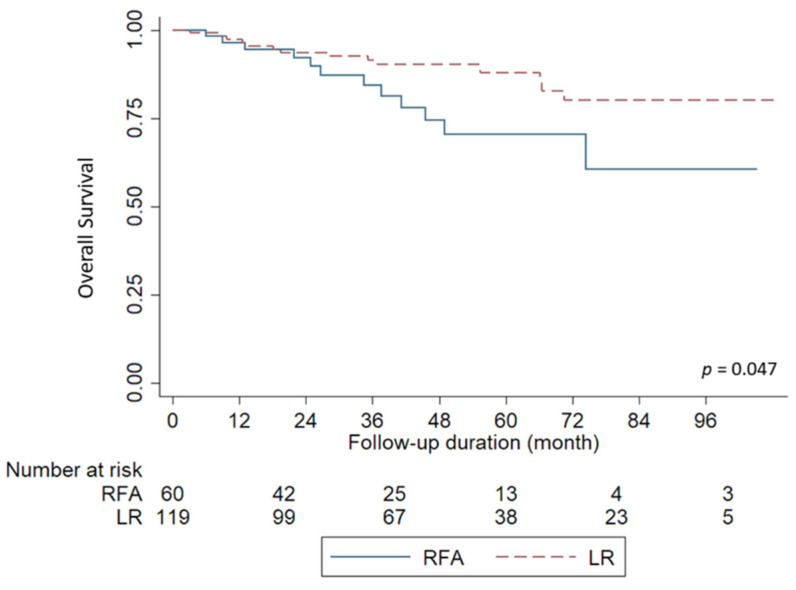
Comparison of overall survival based on the treatment modalities. Liver resection (LR) had a significantly better overall survival than radiofrequency ablation (RFA) for patients (*p* < 0.047).

Twelve of 60 patients (20%) in the RFA group and 14 of 119 (11.7%) LR patients died of HCC during follow-up. The Kaplan-Meier survival curves for OS are shown in Figure 2. The 1-,2-, and 5-year OS rates in the RFA and LR groups were 96.4%, 92.2%, and 71.5% *versus* 97.3%, 93.6%, and 87.8%, respectively (*p* = 0.047).

### 3.3. Risk Factors for HCC Recurrence

Further detailed analyses of prognostic factors for HCC recurrence are summarized in Table 2. Among the clinical variables, age >50 years, liver cirrhosis, multiple tumors, and RFA were found to be significant risk factors in the univariate analysis. Subsequently, multivariate analysis indicated that liver cirrhosis (hazard ratio, HR, 2.130; 95% confidence interval, CI: 1.113–4.075; *p* = 0.02), multiple tumors (HR 3.209; 95% CI: 1.794–5.738; *p* < 0.01), and RFA (HR 1.805; *p* = 0.01) were significantly associated with HCC recurrence.

### 3.4. Small HCC (<2 cm)

**Figure 3:**
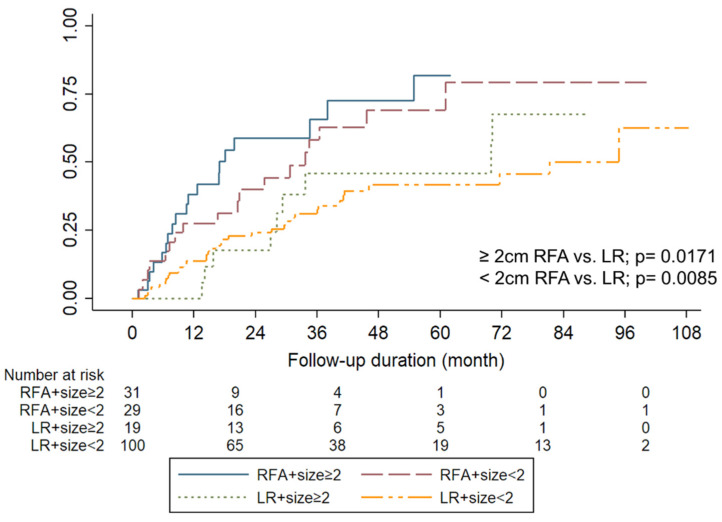
Cumulative recurrent rates of patients according to tumor size and treatment modalities. The cumulative incidence of HCC recurrence was significantly higher for patients who underwent radiofrequency ablation (RFA) in both tumor size ≥2 cm and <2 cm as compared with that of liver resection (LR).

Generally, HCCs < 2 cm are categorized into stage 0, according to the BCLC staging system; thus, outcomes related to small HCCs were further analyzed. The cumulative recurrence rate with respect to the tumor diameter and treatment modalities is illustrated in Figure 3. The 1-, 2-, and 5-year cumulative recurrence rates were 27.5%, 39.9%, and 69.0%, respectively, in the RFA group, and 13.6%, 24.2%, and 41.4%, respectively, in the LR group HCC diameter < 2 cm (*p* = 0.008). Additionally, the 1-, 2-, and 5-year cumulative recurrence rates were 38.2%, 58.9%, and 81.4%, respectively, in the RFA group, and 0%, 17.6%, and 45.9%, respectively, in the LR group for HCC diameter ≥2 cm (*p* = 0.017).

### 3.5. Liver Resection by Laparoscopy or Open Method

**Figure 4:**
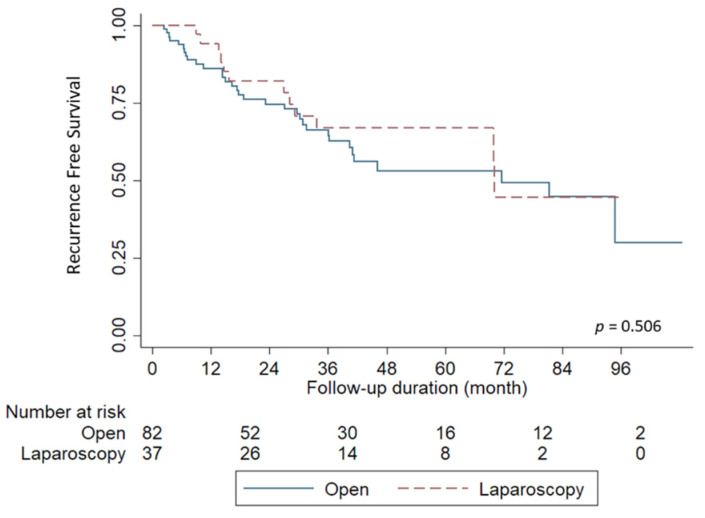
Comparison of recurrence free survival. Patients with liver resection showed comparable outcomes between laparoscopic approach and conventional open method (*p* = 0.506).

Of the 119 patients who underwent LR, 82 received the conventional open method and 37 underwent the laparoscopic approach (Table 3). Overall, 33 of 82 patients (40.2%) who underwent the open method experienced HCC recurrence; 12 of 37 patients (32.4%) in the laparoscopic group had HCC recurrence. The Kaplan-Meier survival curves of RFS, with respect to the operation method, are shown in Figure 4. The open and laparoscopic approaches showed no significant difference in terms of RFS (*p* = 0.506). The median time to HCC recurrence was 17.6 months (range, 2.4–94.8) in the open group and 21.3 months (range, 8.9–70.1) in the laparoscopy group. The 1-, 2-, and 5-year RFS rates in the laparoscopic and conventional open LR groups were 94.1%, 82.0%, and 66.9% versus 86.1%, 74.6%, and 53.1%, respectively.

## 4. Discussion

This study showed that LR provided longer RFS than RFA when treating HCC at LLS; the outcome of LR related to operative methods showed that the laparoscopic approach is comparable to the open method. In addition, the presence of liver cirrhosis, multiple tumors, and treatment with RFA were identified as significant factors related to HCC recurrence after curative treatment.

The advantage of LR in terms of RFS was observed after surgery and lasted for more than 5 years in our series. However, some studies showed that the differences in recurrence appeared 1–2 years after treatment, and some of these showed no significant differences [1,3,18,19,20,21]. This might be explained by selection bias before treatment since the inclusion criteria in each study varied. Recently, a meta-analysis including seven randomized control trials (RCTs) and 18 matched non-RCTs showed superiority of LR in terms of RFS and recurrence incidence [22]. However, none of these studies focused on HCC at LLS. Theoretically, LLS has a thinner hepatic parenchyma that is relatively easier to transect and control bleeding during operation, as compared with other hepatic segments. As such, LR for HCC at LLS was considered a relatively simple and safe procedure, either by open surgery or laparoscopy [3,11].

The presence of cirrhosis, multiple tumors, and RFA were found to be risk factors for HCC recurrence in this study. Two of the factors were related to primary conditions; the only controllable factor was the choice of treatment modality, especially for patients who were suitable for both treatment modalities. Although LR showed superior survival benefit compared to RFA for HCC at LLS, it was not shown to be an independent factor for RFS in other series [1,18,21]. Generally, tumor behavior and status of the patient’s liver remain the most important factors affecting HCC recurrence. The treatment modalities would only play a minor role and rarely impact the outcome of patients. Nonetheless, patients who received LR were younger and had less cirrhosis than those who received RFA. In line with previous studies, the severity of cirrhosis has been reported as a risk factor for HCC recurrence after curative treatment [23,24]. Additionally, the ratio of thrombocytopenia, which is a sign of portal hypertension in cirrhotic patients, was higher in the RFA group. Therefore, the higher recurrence rate after RFA in our series could also be partially explained by the higher incidence of cirrhosis. Moreover, the correlation between treatment modality and recurrence pattern might also affect the outcome of patients with HCC [25,26]. The pattern of HCC recurrence has a significant impact on post-recurrence survival, in which patients with distant metastasis and systemic recurrence are associated to a poorer prognosis.

Additionally, a retrospective study comparing RFA and LR revealed the superior outcome of LR for HCC ≤ 3 cm at LLS in terms of RFS and OS [12]. In that study, the RFS and OS curves of LR and RFA were similar in the first 2 years, which is different from the current study. It also focused on solitary HCC with a tumor size ≤3 cm, whereas this study included patients with larger and multiple tumors. According to our findings, better outcomes are still observed in the LR group, even in the presence of larger and multiple tumors. In fact, few patients received RFA for HCC larger than 5 cm, in consideration of patients’ desires in this study. As such, RFA might be not suitable for large HCCs located at left lateral segment characterized by relatively small liver volume with thin shape. Although RFA has been shown to be an effective and safe procedure for large HCCs [27,28], similar scenarios might be not completely fit for left lateral segment HCCs. Additionally, the combination of transcatheter arterial chemoembolization with RFA might be able to provide a better prognosis for patients with HCC. Although trans-arterial chemoembolization is usually the standard of care for intermediate stage HCC, either trans-arterial embolization or trans-arterial chemoembolization could also be considered in combination with RFA to improve the outcome for HCC patients [29,30].

Generally, both RFA and LR are listed as curative treatment for early stage HCC according to current diagnostic algorithm and clinical practice guideline [16,17]. However, treatment options should depend on thoroughly consideration of the patient’s physical condition, the tumor’s characteristics, and underlying liver cirrhosis. The advantage of RFA over LR is its lower invasiveness and chance of operative risk. However, this advantage might be diminished in the era of minimally invasive surgery. Specifically, laparoscopic LR has been rapidly growing during the last decade, and left lateral sectionectomy is one of the most commonly performed laparoscopic LR procedures [31]. In addition, the study also showed that the outcome of laparoscopic LR was comparable to that of patients who received conventional open LR. Therefore, laparoscopic LR could be considered a standard practice for HCC at LLS, as is the consensus from the “Louisville Statement” [32,33]. Furthermore, laparoscopic LR has also been reported to provide better tumor control and superior outcomes in patients with early-stage HCC than RFA treatment [34]. Although laparoscopic LR is still more invasive than RFA, the discrepancy could also be compensated for by better survival outcomes of HCC patients.

In conclusion, this study might be limited by its retrospective nature and the relatively small number of patients. Although HCC at LLS might not be able to represent the whole population of patients with HCC, a few remarkable observations from the study might be helpful in developing therapeutic strategies for patients with HCC. Generally, outcomes related to early HCC at LLS are satisfactory after curative treatment. However, LR could be considered a priority treatment if patients have no contraindications for surgery. Moreover, laparoscopic left lateral sectionectomy showed comparable results to the open method. Therefore, it may also be recommended as a preferred option in the era of minimally invasive surgery.

## Figures and Tables

**Table 1:** Demographic features of patients undergoing curative treatment for hepatocellular carcinoma at left lateral segment.

Characteristic	Overall (*n* = 179)	RFA (*n* = 60)	LR (*n* = 119)	*p* Value
Age, median (range) <50 years ≥50 years	60.1 (36.5–90.9) 8 (4.5) 171 (95.5)	71.7 (50.2–90.6) 0 (0) 60 (100.0)	64.97 (36.54–90.9) 8 (6.7) 111 (93.3)	0.0011 0.0399
Sex Male Female	123 (68.7) 56 (31.3)	35 (58.3) 25 (41.7)	88 (74.0) 31 (26.0)	0.0334
Cirrhosis No Yes	47 (26.4) 132 (73.6)	3 (5.0) 57 (95.0)	44 (37.3) 75 (62.7)	<0.0001
Etiology HBV HCV HBV+HCV	78 (43.6) 48 (26.8) 7 (3.9)	18 (30.0) 24 (40.0) 2 (3.3)	60 (50.5) 24 (20.1) 5 (4.2)	0.0075
Others	46 (25.7)	16(26.7)	30 (25.2)	
Tumor Number, median (range)	1 (1–3)	1 (1–3)	1.00 (1.0–3.0)	0.2987
Tumor size (cm), median (range)	2.3 (0.6–9.1)	2.0 (0.9–5.2)	2.7 (0.6–9.1)	0.0113
TNM Stage Stage I Stage II	118 (65.9) 35 (19.5)	51 (85.0) 7 (11.7)	67 (56.3) 28 (23.5)	0.0129
Platelet, median (range) <10 × 10^3^/μL ≥10 × 10^3^/μL	147.0 (30.0–536.0) 43 (24.1) 136 (75.9)	112.0 (30.0–381.0) 26 (43.3) 34 (56.7)	168.0 (38.0–536.0) 17 (14.3) 102 (85.7)	<0.0001 <0.0001
AFP (ng/mL), median (range)	21.2 (1.7–136275.9)	23.7 (2.3–21075.7)	20.1 (1.7–136275.9)	0.2514
HCC recurrence	81(45.3)	36 (61.6)	45 (37.8)	0.0025
* HCC recurrent pattern				0.0021
Local intrahepatic	7 (8.6)	7 (19.4)	0 (0)	
Distant intrahepatic	67 (82.8)	25 (69.4)	42 (93.3)	
Systemic metastasis	7 (8.6)	4 (11.2)	3 (6.7)	
Complications (≥grade II)	11(6.2)	3 (5.0)	8 (6.7)	0.6519
Hospital stay, days, median (range)		3 (2–9)	7 (4–38)	<0.0001

RFA: radiofrequency ablation, LR: liver resection, TNM: Tumor-Node-Metastasis, HBV: hepatitis B virus, HCV: hepatitis C virus, * represents the percentage among HCC recurrence, AFP: α-fetoprotein.

**Table 2:** Univariate and multivariate analyses of clinicopathological factors affecting HCC recurrence in patients after curative treatment.

Variables	Overall (*n* = 179)	Recurrence (*n* = 81)	Crude HR (95% CI)	*p* Value	Multivariate HR (95% CI)	*p* Value
Age, median(range) <50 years ≥50 years	60.1 (36.5–90.9) 8 (4.5) 171 (95.5)	68.6 (38.6–90.8) 2 (2.5) 79 (97.5)	1.024 (1.004–1.045) 1.0 2.402 (0.589–9.794)	0.0197 0.2216	1.022 (1.000–1.046)	0.0550
Sex Male Female	123 (68.7) 56 (31.3)	62 (76.5) 19 (23.5)	1.0 0.750 (0.449–1.253)	0.2714		
Cirrhosis No Yes	47 (26.4) 132 (73.6)	12 (14.8) 69 (85.2)	1.0 2.353 (1.274–4.343)	0.0062	2.130 (1.113–4.075)	0.0224
Etiology HBV HCV HBV+HCV	78 (43.6) 48 (26.8) 7 (3.9)	40 (49.4) 21 (25.9) 2 (2.5)	1.0 1.055 (0.619–1.796) 0.636 (0.153–2.635)	0.8448 0.5322		
Others	46 (25.7)	18 (22.2)	0.855 (0.494–1.478)	0.5741		
Tumor Number	1 (1–3)	1 (1–3)	2.909 (1.658–5.106)	0.0002	3.209 (1.794–5.738)	<0.0001
Tumor size (cm)	2.3 (0.6–9.1)	2.4 (0.8–8.0)	1.011 (0.870–1.175)	0.8823		
TNM Stage Stage I Stage II	118 (65.9) 35 (19.5)	53 (65.4) 15 (18.5)	1.0 0.867 (0.489–1.539)	0.6262		
Platelet <10 × 10^3^/μL ≥10 × 10^3^/μL	43 (24.1) 136 (75.9)	134.00 (30.0–531.0) 20 (24.7) 61 (75.3)	1.0 0.727 (0.436–1.210)	0.2198		
AFP (ng/mL)	21.2(1.7–136275.9)	32.5 (1.7–136275.9)	1.000 (1.000–1.000)	0.0623		
Treatment RFA LR	60 (33.5) 119 (66.5)	36 (37.8) 45 (61.6)	2.426 (1.559–3.776) 1.0	<0.0001	1.804 (1.127–2.886)	0.0140

HCC: hepatocellular carcinoma, HBV: hepatitis B virus, HCV: hepatitis C virus, TNM: Tumor-Node-Metastasis, AFP: α-fetoprotein, RFA: radiofrequency ablation, LR: liver resection, HR: hazard ration, CI: confidence interval.

**Table 3:** Comparison of clinical characteristics according to liver resection approach for patients with HCC.

Characteristic	Laparoscopy (*n* = 37)	Open (*n* = 82)	*p* Value
Age, median (range) <50 years ≥50 years	64.4 (36.5–90.9) 2 (5.0) 35 (95.0)	65.1 (39.9–90.1) 6 (7.32) 76 (92.68)	0.9138 0.6999
Sex Male Female	30 (81.1) 7 (18.9)	58 (70.7) 24 (29.3)	0.2338
Cirrhosis No Yes	12 (32.4) 25 (67.6)	33 (39.0) 49 (61.0)	0.3163
Etiology HBV HCV HBV+HCV	21 (56.8) 8 (21.6) 1 (2.7)	39 (47.6) 16 (19.5) 4 (4.8)	0.7942
Others	7(18.9)	23(28.1)	
Tumor Number, median (range)	1.0 (1.0–2.0)	1.0 (1.0–3.0)	0.9077
Tumor size (cm), median (range)	2.6 (0.5–7.8)	3.2 (0.9–9.10)	0.1161
TNM Stage Stage I Stage II	26 (70.3) 11 (29.7)	49 (59.8) 33 (40.2)	0.2736
Platelet, median (range) <10 × 10^3^/μL ≥10 × 10^3^/μL	167.0 (95.0–312.0) 2 (5.4) 35 (94.6)	179.0 (38.0–536.0) 15 (18.3) 67 (81.7)	0.36 0.3319
AFP (ng/mL), median (range)	8.2 (1.7–24529.2)	29.9 (2.2–136275.9)	0.08

HCC: hepatocellular carcinoma, HBV: hepatitis B virus, HCV: hepatitis C virus, TNM: Tumor-Node-Metastasis, AFP: α-fetoprotein. Number in the parenthesis represents percentage of each subgroup.

## Data Availability

All data were included in the study.
